# Enhanced emotion regulation capacity and its neural substrates in those exposed to moderate childhood adversity

**DOI:** 10.1093/scan/nsv109

**Published:** 2015-09-04

**Authors:** Susanne Schweizer, Nicholas D. Walsh, Jason Stretton, Valerie J. Dunn, Ian M. Goodyer, Tim Dalgleish

**Affiliations:** ^1^Medical Research Council Cognition and Brain Sciences Unit, Cambridge, UK,; ^2^University of East Anglia, School of Psychology, Norwich, UK,; ^3^Department of Psychiatry, University of Cambridge, Cambridge, UK, and; ^4^Cambridgeshire and Peterborough NHS Foundation Trust, Cambridge, UK

**Keywords:** childhood adversity, emotion regulation, resilience, adolescence, amygdala

## Abstract

Individuals exposed to childhood adversities (CA) present with emotion regulation (ER) difficulties in later life, which have been identified as risk and maintenance factors for psychopathologies. However, it is unclear if CA negatively impacts on ER capacity *per se* or whether observed regulation difficulties are a function of the challenging circumstances in which ER is being deployed. In this longitudinal study, we aimed to clarify this association by investigating the behavioral and neural effects of exposure to common *moderate* CA (mCA) on a laboratory measure of ER capacity in late adolescence/young adulthood. Our population-derived samples of adolescents/young adults (*N* = 53) were administered a film-based ER-task during functional magnetic resonance imaging that allowed evaluation of ER across mCA-exposure. mCA-exposure was associated with *enhanced* ER capacity over both positive and negative affect. At the neural level, the better ER of negative material in those exposed to mCA was associated with reduced recruitment of ER-related brain regions, including the prefrontal cortex and temporal gyrus. In addition mCA-exposure was associated with a greater down-regulation of the amygdala during ER of negative material. The implications of these findings for our understanding of the effects of mCA on the emergence of resilience in adolescence are discussed.

## Introduction

Emotion regulation (ER) abilities develop throughout childhood, adolescence and into adulthood (Thompson and Meyer, 2007). Relative impairments in ER are a marker of maladaptive social and occupational functioning, and a risk factor for psychopathology (Aldao *et al.*, 2010). Understanding the etiology of problems with ER is therefore of critical importance (Campbell-Sills and Barlow, 2007).

One contributor appears to be significant early-life stress, whether it be severe physical or sexual abuse (Pechtel and Pizzagalli, 2010), or far subtler forms of adversity such as maternal insensitivity (Feldman *et al.*, 2004). These detrimental effects of adversity on ER are typically measured through either self-report or observation within the family environment (Morris *et al.*, 2007) thus providing an index of ER difficulties in day-to-day life. However, it is unclear whether poorer ER on such indices reflects adversity-related deficits in ER *capacity per se* or, instead, the significant challenges posed to ER by the aversive contexts themselves, such that anyone would experience ER difficulties in similar circumstances.

This is an important distinction because, plausibly, some forms of mild-to-moderate childhood adversity (CA) could augment underlying ER capacity by providing more opportunities to learn and practice ER skills, as evidenced by studies from the rodent and non-human primate literature (Parker and Maestripieri, 2011), and preliminary work in humans (Fergus and Zimmerman, 2005; Seery *et al.*, 2013). It therefore seems critical to extend our understanding of the interplay between CA and ER by elucidating the effects of CA on ER capacity itself.

Any effects of moderate childhood adversity (mCA) on ER capacity are likely to be mediated by changes in brain structure and function (Belsky and de Haan, 2011). A substantive body of neuroimaging literature has investigated the effects of CA on the brain (Whittle *et al.*, 2013), with numerous studies examining the association between CA and aspects of emotion processing other than ER (e.g. van Harmelen *et al.*, 2010; Tottenham *et al.*, 2011). Preliminary evidence from both structural and functional imaging research suggests two key sites of action for the association between CA and ER—the amygdala and the PFC (Cohen *et al.*, 2013; McEwen and Morrison, 2013) (see Supplementary Materials for the effects of CA on amygdala and PFC development). To date, the only neuroimaging study to have examined the neural substrates of the link between early CA (i.e. childhood poverty and chronic stress) and ER showed that exposure to poverty at age 9 was associated with reduced (compared with non-CA exposed individuals) activation in the frontopartietal control network during the reappraisal of aversive images, relative to simply mainting the emotions elicited by these negative images (Kim *et al.*, 2013). The study further showed a negative association between childhood income and amygdala activation.

As mentioned above, there is, however, an emerging body of literature suggesting that in some instances adversity can promote resilience, wherein strong ER skills are considered fundamental to resilience (Buckner *et al.*, 2003, 2009; Fergus and Zimmerman, 2005; Cicchetti, 2010; Crowell *et al.*, 2013). Neurobiologically, resilience has typically been investigated in terms of endocrinological and immunological (e.g. corticotropin-releasing hormone mRNA concentrations, dopamine, gonadal steroids), and peripheral (e.g. heart rate variability) functioning (Charney, 2004; Zautra *et al.*, 2008). Human research, however, lags behind in the understanding of the neuroanatomical and functional correlates of resilience (Charney, 2004). In a notable exception, Cisler *et al.* (2013) show reduced connectivity in the ventrolateral PFC and dorsal ACC during the regulation of emotions to negative pictures in individuals that experienced early life stress but never developed depression compared with those with a history of CA and depression. Trait resilience has also been associated with greater neural flexibility (faster return to baseline insula activation) to neutral stimuli in threat contexts (Waugh *et al.*, 2008).

In sum, then ER capacity may convey either a risk or resilience factor following mCA, with its neural correlates likely to be expressed in terms of PFC and/or amygdala activation. The only study, to our knowledge, to have directly investigated the effects of early CA on ER and its neural correlates (Kim *et al.*, 2013), provides an important first step toward a better understanding of their association. However, the study failed to control for a number of important potential confounding factors, which may account for their results (e.g. current mood, previous psychiatric history, parental psychiatric history, genetic risk factors).

In this study, we therefore investigated whether prior mCA was associated with either increased or decreased ER capacity and the neural substrates of any such effects. To investigate the effects of mCA on ER capacity *per se* we used a laboratory measure of ER capacity that is decontextualized from the individual’s personal circumstances obtained by evaluating response to standardized emotive film clips (Schweizer *et al.*, 2013). This measure of ER arguably elicits distress more akin to that experienced in everyday life compared with emotive reactions elicited by negative pictures (used in the studies investigating both risk and resilience of CA on ER reviewed here). We further chose mCA as this seemed a more likely context for the enhancement of ER capacity, relative to severe early life stress (Zolkoski and Bullock, 2012). mCA is relatively common, with some 1-in-4 children exposed to a moderate degree of sub-optimal family environments (Dunn *et al.*, 2011). Using a longitudinal design, we therefore used this approach to measure ER capacity in individuals at the cusp between adolescence and early adulthood (age range 19–21 years) previously exposed to mCA. The opportunity to recruit from the broader ROOTS cohort (described below; Goodyer *et al.*, 2010) also allowed us to match participants across CA for a number of confounding factors that have potentially influenced findings in the literature relating adversity to behavioral and neural aspects of emotion functioning.

## Materials and methods

The study was approved by the Cambridgeshire Research Ethics Committee. All participants provided written informed consent.

### Participants

Inclusion criteria were as follows: membership of the ROOTS cohort (*N* = 1243; Goodyer *et al.*, 2010); normal or corrected-to-normal vision; and English speaking. Exclusion criteria were as follows: any history of neurological trauma resulting in loss of consciousness; current psychotropic medication use; current neurological disorder; current DSM-IV (American Psychiatric Association, 2000) Axis 1 psychiatric disorder (we opted for a current psychiatric diagnosis-free sample to avoid complicating the inferences made from the present results as a concomitant or consequence of any pathological mental state, rather than prior mCA); presence of metal in the body; specific learning disability, and IQ <85 on the Wechsler Abbreviated Scale of Intelligence (Wechsler, 1997).

The 53 participants in this study were a subsample selected from the ROOTS cohort based on their CA classification on the Cambridge Early Experience Interview (CAMEEI; see Supplementary Materials and Methods section), fully completed by 1143 (92%) of this sample at age 14 (Dunn *et al.*, 2011). Participants were included based on their categorization on the CAMEEI as being exposed (mCA+ ; *n* = 23) or not exposed (mCA−; *n* = 30) to mCA before the age of 11 years. In this investigation, mCA was defined as exposure to significant (moderate-to-severe) family discord, occasional physical violence between family members, lack of affectionate warmth between family members or severe lack of communication between family members, in the absence of either probable or confirmed physical or sexual abuse. For an overview of the distribution of mCA type and severity, see Table 1. Such mCA is associated with a moderate increase in risk (odds ratio 1.3–3.9) for emotional and behavioral disorders by 14 years of age in the overall ROOTS cohort (Dunn *et al.*, 2011). Our design therefore allowed us to prospectively investigate the effects of mCA on ER capacity measured in late adolescence/early adulthood.

**Table 1.** Characteristics of participants exposed to moderate childhood adversity (mCA+; n = 23) derived from the CAMEEI

CAMEEI variable	
Age of exposure (years; M (s.d.))	5.1 (3.3)
Duration CA (months; M (s.d.))	27.0 (17.2)
Sexual abuse (none, possible, probable)	100%, 0%, 0%
Emotional abuse (none, possible, probable)	87%, 0, 13%
Physical abuse (none, possible, probable)	91%, 9%, 0%
Inter-parental conflict (none, possible, probable)	0%, 0%, 100%

The longitudianal nature of the sample further allowed us to match the groups for a number of potentially counfounding factors. Specifically, participants were group-matched on age, gender, current mood status (based on mood assessed on average 2 weeks prior to the present scan date), socioeconomic status (SES), IQ and 5-HTTLPR genotype (l/l *vs* s/s; see Supplementary Materials and Methods section for DNA collection, extraction and analysis details). We matched on age because brain maturation in the regions involved in ER in typical development undergoes significant changes in late adolescence and early adulthood—the age range of the ROOTS cohort (Pitskel *et al.*, 2011; McRae *et al.*, 2012). Groups were matched for gender because women report using more ER strategies, and because different ER strategies are associated with psychopathology in women and men (e.g. more rumination and alcohol use, respectively; Nolen-Hoeksema, 2012). We matched for mood status because of its influence on dispositional use of ER strategies (Kokkonen and Pulkkinen, 2001; Gross and Thompson, 2007). The groups were further matched for SES as CA in general is more prevalent in children from lower SES backgrounds (Herrenkohl and Herrenkohl, 2007) and lower childhood SES has been linked to later deficits in ER in adults (Kim *et al.*, 2013). We chose to additionally match the groups on IQ to control for the potentially confounding effects of intelligence on task adherence and comprehension (Duncan, 2010), as well as the fluency with which participants could generate reappraisals during the ER task (Nusbaum and Silvia, 2011). We matched for 5-HTTLPR polymorphism as previous studies have shown that carriers of the short allele show attentional biases toward negative information and greater reactivity to emotional information as measured by amygdala activity (Hariri and Holmes, 2006; Canli and Lesch, 2007; Canli *et al.*, 2009).

### The ER task (ERT)

**Fig. 1.**
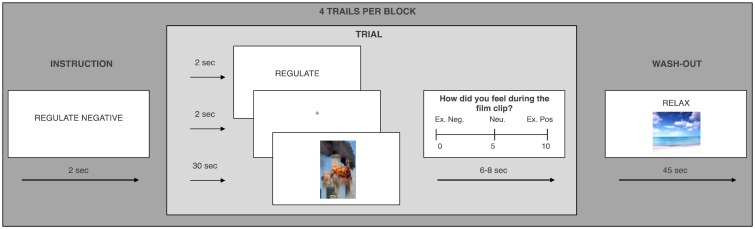
ERT design. A sample block in the Negative Regulate condition is shown. The blocks were identically structured across conditions except that the neutral clips were not followed by a 45-s washout clip designed to return participants emotions back to baseline. Emotions were rated on a scale ranging from ‘0’ = Extremely negative, through ‘5’ = Neutral, to ‘10’ = Extremely positive.

The ERT (Figure 1) assesses participants’ ability to downregulate (ER) their affective responses to pleasant or aversive film footage (Goldin *et al.*, 2008; Schweizer *et al.*, 2013). The task presents participants with a series of 30-s film clips in five experimental conditions. These comprise three Attend only conditions—attending to neutral (Attend Neutral), aversive (Attend Negative) or pleasant films (Attend Positive)—without engaging in any attempts to reduce the affective response elicited by the stimuli*.* Critically, these are supplemented by two Regulate conditions where participants viewed negative (Regulate Negative) or pleasant (Regulate Positive) films while trying to effortfully downregulate emotional responses to the films by actively reappraising their content (Gross, 2002). Each condition was presented twice in the MRI scanner in a blocked design, with four trials in each block. Emotional blocks were followed by 45-s washout clips, to return mood to pre-stimulus baseline. Films were randomized across the Attend and Regulate conditions separately for each participant and the presentation order of condition was pseudo-randomized always starting with a neutral block and ending with a positive block. To index ER capacity, we computed a Regulation index for Negative and Positive affect separately. The indices were computed by subtracting Attend Negative/Positive emotion ratings from Regulate Negative/Positive ratings.

We further took advantage of the task’s capacity to generate measures of emotional reactivity. Separate Negative and Positive Emotional Reactivity Indices were computed by subtracting emotion ratings during the Attend Neutral condition from, respectively, emotion ratings in the Attend Negative and in the Attend Positive conditions. Ratings from the Neutral condition were subtracted to control for individual differences in scale use and in baseline affect. This allowed us to evaluate the extent to which any differences across our two groups in terms of ER capacity were a function of underlying differences in emotional reactivity.

Before performing the task in the scanner, participants saw three practice films in each condition and were asked to verbalize their maintenance or regulatory strategies after each film clip.

### Neuroimaging analyses

For image acquisition parameters, see Supplementary Materials and Methods section. Imaging analyses were performed with a full-factorial design at this second-level with mCA-exposure as an independent group factor, and for the ER and reactivity analyses, condition was added as an additional independent factor. The regions of interest (ROIs) for our ER imaging analyses were selected based on a meta-analysis of reappraisal as an ER strategy (Buhle *et al.*, 2014). We chose the meta-analysis of reappraisal as this was the strategy that participants were instructed to use to downregulate their emotions in our ER task. That is, we asked participants to change their levels of distress by changing the way they thought about the content of the film clip. We defined 10-mm spheres around the peak activations for each of the clusters identified in the meta-analysis of reappraisal using MarsBaR (Brett *et al.*, 2002), with the exception of the two superior parietal gyrus clusters as this region was not fully included in the field of view due to a focus of the orientation in this study to include the medial and anterior temporal regions and the amygdala, especially. We therefore extracted mean activation for the following ROIs (Supplementary Figure S1): right inferior frontal gyrus (peak coordinates: 51/15/48), bilateral middle frontal gyrus (left: −33/3/54, right: 60/24/3), right medial frontal gyrus (9/30/39), left middle temporal gyrus (−51/−39/−3) and bilateral amygdala (left: −18/−3/−15, right: 30/−3/−15).

Extracted values were analyzed using SPSS version 22 (IBM Corp., 2013). We entered these extracted mean levels of BOLD activation for each ROI in a series of repeated measures analyses of variance with two levels: ER task condition and group (mCA+, mCA−). We used an unadjusted the level of *α* = 0.05 to examine our ROIs because the extraction of average BOLD activation across a 10-mm sphere ROI is already a conservative analytic approach (as activation in only a subset of voxels within the sphere might be sensitive to our experimental effects), that biases the analyses in favor of the null hypothesis (Poldrack, 2007). Furthermore, identification of the ROIs from meta-analytic data based on 48 prior studies (Buhle *et al.*, 2014) provides us with clear *a priori* hypotheses for each region, as part of a reappraisal neural network. For any ROIs showing a significant differential effect of ER condition across group, we investigated their correlation with behavioral performance on the ERT.

To investigate the functional connectivity of any ROIs showing a significant condition by mCA interaction, we deconvolved the BOLD time series for each participant to estimate a ‘neuronal time series’ (Gitelman *et al.*, 2003). The psychophysiological interaction term (PPI regressor) was calculated as the element-by-element product of the ROIs’ neuronal time series and a vector coding for the main effect of the condition (thresholded at *P* < 0.001 uncorrected). This product was re-convolved by the canonical hemodynamic response function. The model also included the main effects of task convolved by the hemodynamic response function, and the movement regressors as effects of no interest. Participant-specific PPI models were run, and contrast images generated for each condition. These ‘first-level’ contrast images were then entered into the general linear models to assess potential effects of mCA on functional connectivity from the ROIs’ seed regions across condition.

## Results

### Participant characteristics

As anticipated, based on the characteristics of the full ROOTS cohort (Dunn *et al.*, 2011), mCA+ individuals in this study reported significantly more previous psychiatric diagnoses [*F*(1,49) = 5.90, *P* = 0.019, *η*_p_^2 ^= 0.11] and were more likely to have a parent with a history of psychiatric illness (*U* = 228.00, *P* = 0.015), than individuals in the mCA−group (Table 2). Subsequent analyses were therefore conducted with previous psychiatric diagnoses and parental psychiatric history as covariates. However, the patterns of results were identical when these covariates were excluded.

**Table 2.** Sample characteristics as a function of mCA status

	mCA+ (*n* = 23)	mCA− (*n* = 30)
Age^a^—*M* (s.d.)	20.13 (.64)	20.07 (.74)
Gender (women)	12 (52%)	14 (47%)
ACORN (A:B:C:M)	12:5:4:2	18:6:4:2
IQ—*M* (s.d.)	108.72 (7.23)	107.11 (9.40)
MFQ—*M* (s.d.)	12.56 (8.87)	8.70 (7.31)
STAI-T—*M* (s.d.)	32.23 (8.36)	32.76 (8.77)
Previous psychiatric diagnosis	10 (44%)	4 (13%)
Parent with psychiatric history	17 (74%)	12 (40%)
5-HTTLPR polymorphism status (ll:ss)	12:11	16:14

*^a^Age range in both samples 19–21 years. ACORN = A Classification of Residential Neighborhoods code: A = Wealthy achievers/urban prosperity; B = Comfortably off; C = Moderate means/Hard pressed; M = Missing. IQ = total score on the Wechsler Abbreviated Intelligence Scale (**Wechsler, 1997);** MFQ = total score on the Mood and Feelings Questionnaire, a well-validated measure of mood in adolescents (**Angold* et al.*, 1995**); STAI = Spielberger State Trait Anxiety Inventory – Trait score, a reliable measure of trait-anxiety (**Spielberger* et al.*, 1970**). mCA+ = adolescents exposed to mild to moderate childhood adversity. mCA− = adolescents not exposed to childhood adversity.*

### ERT performance

Behavioral performance on the ERT across the whole sample verified that the task was performing as expected—self-reported valence and intensity of emotional reactions during the film clips across the five ERT conditions (i.e. Attend Neutral/Negative/Positive and Regulate Negative/Positive) differed significantly [*F*(1,49) = 129.60, *P* < 0.001, *η*_p_^2 ^= 0.91]. Planned pairwise comparisons revealed (Table 3) that each condition differed significantly (*P* < 0.001) from all other conditions. Specifically, as one would predict, participants overall reported the highest level of negative emotions in the Attend Negative condition followed by Regulate Negative, Attend Neutral, Regulate Positive and Attend Positive.

**Table 3.** Emotionality ratings across the ERT conditions for the whole sample

	M (s.d.)	Attend Negative	Regulate Negative	Attend Positive	Regulate Positive
		*t* (52)	*t* (52)	*t* (52)	*t* (52)
Attend Neutral	6.23 (.35)	18.58	12.43	14.73	4.84
Attend Negative	3.84 (.87)	–	6.05	23.38	16.39
Regulate Negative	4.47 (.90)		–	19.29	10.99
Attend Positive	7.78 (.67)			–	5.33
Regulate Positive	7.04 (.95)				–

**Fig. 2.**
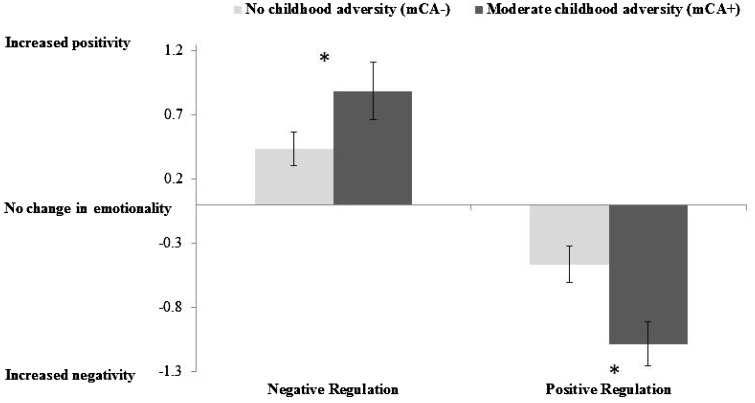
The effect of mCA on emotion regulation. *P < 0.05 indices were computed from emotion ratings across conditions as follows: Negative Regulation = Regulate Negative – Attend Negative; Positive Regulation = Regulate Positive – Attend Positive. Note: for the Positive Regulate Index increased negativity reflects successful regulation as participants were asked to reduce (downregulate) their positive feelings during the positive film clip.

The scores for the individual experimental conditions on the ERT for the two groups separately are presented in Table 4. Figure 2 presents the computed Positive and Negative Regulation indices. As can be seen, in terms of ER capacity, participants in the mCA+ group exhibited a greater ability to downregulate affect on both the Negative Regulation Index [*F*(1,49) = 5.06, *P* = 0.029, *η*_p_^2^ = 0.09] and the Positive Regulation Index [*F*(1,49) = 7.60, *P* = 0.008, *η*_p_^2 ^= 0.13], compared with mCA−participants.

**Table 4.** Emotionality ratings for the different conditions on the ERT for the two mCA groups

	mCA−	mCA+
	M (s.d.)	M (s.d.)
Attend Neutral	6.28 (0.31)	6.16 (0.39)
Attend Negative	3.96 (0.76)	3.68 (1.01)
Regulate Negative	4.39 (0.67)	4.57 (1.14)
Attend Positive	7.65 (0.70)	7.95 (0.60)
Regulate Positive	7.18 (0.86)	6.86 (1.04)

**Fig. 3.**
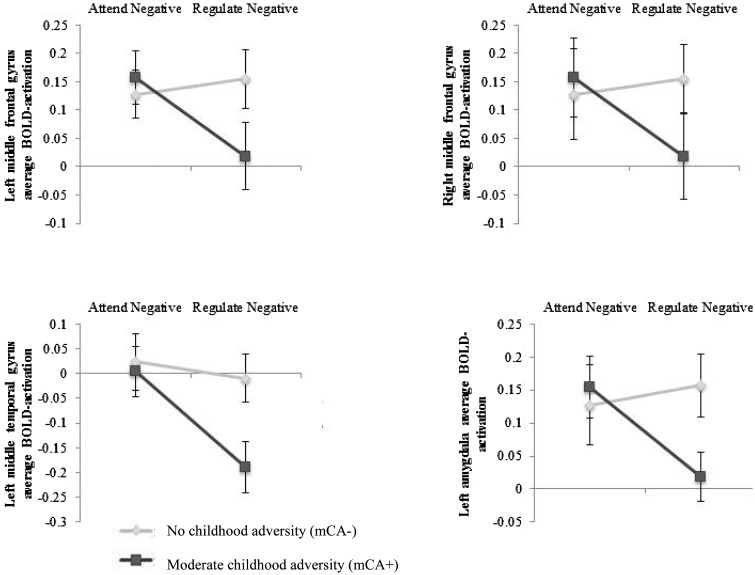
Average blood oxygen level dependent (BOLD) activation in ROIs showing a significant difference in negative ER as a function of mCA status.

In the case of the negative films, these behavioral effects of ER across groups were reflected within the fMRI data (Figure 3; see Table 5 for an overview of mean BOLD activation in all ROIs for each condition across groups).^1^ For the left amygdala ROI there was a significant group by condition interaction [compared with Attend Negative− left: *F*(1,51) = 4.52, *P = *0.038, *η*_p_^2 ^= 0.08, right: *F*(1,51) = 3.70, *P* = 0.060, *η*_p_^2 ^= 0.07]. Compared with the mCA− group mCA+ participants showed a significantly greater reduction in left lateralized activation (with a trend for the right amygdala activation) when downregulating negative affect. The mCA+ group also showed a significantly greater reduction in activation bilaterally in the middle frontal gyrus [left: *F*(1,51) = 4.56, *P* = 0.038, *η*_p_^2 ^= 0.08, right: *F*(1,51) = 4.78, *P* = 0.033, *η*_p_^2 ^= 0.09] and in the left middle temporal gyrus [*F*(1,51) = 4.56, *P* = 0.038, *η*_p_^2 ^= 0.08] during the downregulation of negative affect (relative to Attend Negative) compared with the mCA− group. There were no significant group differences associated with negative ER in the right inferior frontal gyrus [*F*(1,51) = 2.97, *P* = 0.091, *η*_p_^2 ^= 0.06] and left medial frontal gyrus [*F*(1,51) = 2.15, *P* = 0.149, *η*_p_^2 ^= 0.04] ROIs. Interestingly, there were also no significant differential effects of mCA exposure on activation associated with the comparison of the Regulate *vs* Attend Positive conditions in any of the ROIs [*F*s (1,51) .023–1.28, *P*s = 0.880–0.264].^2^

**Table 5.** Mean BOLD activation in all ROIs across conditions for the mCA+ and mCA− groups

ROI	L/R	Attend Neutral		Attend Negative		Attend Positive		Regulate Negative		Regulate Positive	
		M (s.d.)		M (s.d.)		M (s.d.)		M (s.d.)		M (s.d.)	
		mCA−	mCA+	mCA−	mCA+	mCA−	mCA+	mCA−	mCA+	mCA−	mCA+
Inferior frontal gyrus	R	0.25 (0.48)	0.13 (0.56)	0.32 (0.50)	0.33 (0.39)	0.31 (0.51)	0.10 (0.58)	0.30 (0.45)	0.03 (0.54)	0.28 (0.45)	0.11 (0.56)
Middle frontal gyrus	L	0.17 (0.35)	0.18 (0.31)	0.19 (0.22)	0.18 (0.23)	0.19 (0.37)	0.10 (0.31)	0.21 (0.27)	0.01 (0.28)	0.18 (0.27)	0.07 (0.27)
	R	0.14 (0.38)	0.07 (0.34)	0.13 (0.44)	0.25 (0.34)	0.25 (0.41)	0.10 (0.44)	0.20 (0.33)	0.05 (0.36)	0.21 (0.35)	0.16 (0.43)
Medial frontal gyrus	R	0.20 (0.33)	0.16 (0.35)	0.21 (0.36)	0.23 (0.22)	0.28 (0.46)	0.19 (0.28)	0.26 (0.29)	0.13 (0.33)	0.23 (0.32)	0.16 (0.34)
Middle temporal gyrus	L	−0.04 (0.31)	−0.16 (0.31)	0.02 (0.31)	0.00 (0.25)	−0.01 (0.33)	−0.16 (0.33)	−0.01 (0.26)	−0.19 (0.25)	0.07 (0.27)	−0.05 (0.28)
Amygdala	L	0.06 (0.28)	0.03 (0.24)	0.13 (0.32)	0.16 (0.21)	0.11 (0.33)	−0.04 (0.32)	0.15 (0.23)	0.02 (0.18)	0.13 (0.25)	0.04 (0.30)
	R	0.04 (0.33)	−0.02 (0.27)	0.09 (0.33)	0.12 (0.30)	0.11 (0.33)	−0.08 (0.32)	0.10 (0.22)	−0.04 (0.19)	0.16 (0.27)	0.01 (0.30)

For these ROIs that showed statistically significant different patterns of activation across groups in association with negative ER, we investigated the correlations with behavioral performance on the ERT for each mCA group separately. There were no significant correlations within the mCA− group (*r*s = 0.03–0.025, *P*s = 0.88–0.21). However, the mCA+ group showed a statistically significant association between activation in the right amygdala during ER (Regulate Negative – Attend Negative) of negative films and the Negative Regulation Index (*r* = 0.49, *P* = 0.025). None of the other ROIs showed a statistically significant association (*r*s = 0.19–0.34, *P*s = 0.42–0.13). However, this is likely to be a power issue as the magnitude of these association indicates a moderate effect.

Finally, we examined whether the differential behavioral and neural effects of mCA on ER capacity were likely to be a function of underlying differential emotional reactivity across the groups. Analysis of the behavioral data showed that there were no significant differences between mCA groups on either the Positive [*F*(1,49) = 2.03, *P* = 0.16 or Negative Reactivity Indices (*F*(1,49) = 1.08, *P* = 0.302].^3^ Mirroring these behavioral findings, there were no significant differences in average BOLD activation during the Attend Negative/Positive *vs* Attend Neutral conditions across the mCA groups for any of our ROIs [see Table 5; *F*s(1,51) = 0.012–2.01, *P*s = 0.913–0.162].

#### Functional connectivity

**Fig. 4.**
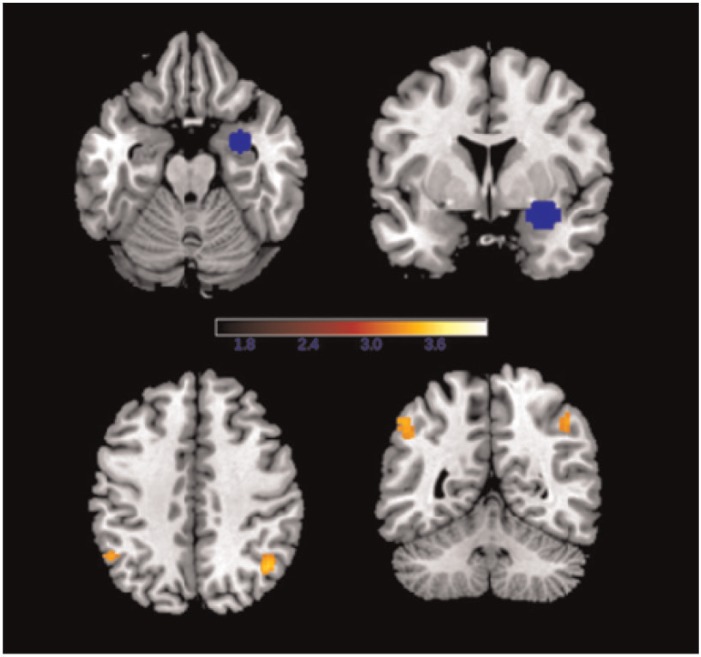
The right amygdala seed ROI (top) and the bilateral inferior parietal clusters to which the mCA+ group showed reduced connectivity compared with the mCA− group during Regulate Negative relative to Attend Negative.

Due to the significant differential involvement of the amygdala, middle frontal and middle temporal gyri in negative ER across the two groups, we conducted functional connectivity analyses using PPI with these ROIs as the seed regions comparing the Regulate Negative *vs* Attend Negative conditions across the groups. The bilateral middle frontal gyrus and left middle temporal gyrus ROIs showed no differential connectivity, nor did the left amygdala. However, when downregulating effect to negative films *vs* simply attending to negative films, the mCA+ group showed reduced functional connectivity from the right amygdala to the bilateral inferior parietal cortex (left: *k* = 57, *z* = 3.28, *−*52/−54/46, right: *k* = 98, *z* = 3.44, 44/−60/44) compared with the mCA− group (Figure 4).

## Discussion

We investigated the effects of mCA on adolescents’/young adults’ ER capacity measured using a laboratory-based film-viewing task. The behavioral data revealed that individuals with a history of mCA were able to regulate their affective responses to both negative and positive emotive film clips more successfully compared with their non-mCA-exposed peers.

The fMRI findings, in the case of negative emotive material, were consistent with the behavioral data. Those exposed to mCA showed reduced recruitment of the middle frontal and temporal gyri when downregulating negative affect—these are regions previously shown to be critical to ER success (Wager *et al.*, 2008; Buhle *et al.*, 2014). Those exposed to mCA also showed reduced amygdala activation during negative downregulation—a reliable neurobiological index of reduced experienced distress (Phan *et al.*, 2003). Finally, the extent of the reduction in (right) amygdala activation in the mCA group in the context of negative films was significantly correlated with greater ER success. This combination of reduced amygdala activation and reduced recruitment of frontal brain regions linked to top-down control when downregulating negative affect suggests that the neural matrix underpinning the more successful ER in negative contexts associated with mCA may be operating more efficiently (Poldrack, 2000). Importantly, there was no suggestion that these ER effects across groups were due to any differences in underlying emotional reactivity as there was no significant effect of mCA-exposure on either behavioral or neural measures of either positive or negative reactivity.

Interestingly, the mCA-exposed participants also showed reduced connectivity during negative ER between the right amygdala and the inferior parietal cortex, an area that has been reported to be involved in visuospatial processing and mental imagery (Cavanna and Trimble, 2006). Mental imagery in turn has been shown to be a very effective ER skill used in the treatment of psychopathology, especially for the symptoms of affective dysregulation (Holmes and Hackmann, 2004; Holmes and Mathews, 2010). Again a reduction in connectivity between those regions may signal more automated use of regulatory strategies.

Taken together, the behavioral and neural findings suggest that mCA is associated with an enhanced capacity for ER in a laboratory setting. This is in contrast with the only other study investigating the neural substrates and association between ER capacity and early CA in young adults (mean age 24 years; Kim *et al.*, 2013), who showed reduced activation in the frontoparietal control network to be associated with worse ER capacity in those exposed to early childhood poverty. There are some noticeable differences between the studies: Importantly, in this study individuals with and without mCA exposure were matched for a number of important confounds (including 5-HTTLPR polymorphism, mood, current diagnosis-free status, sociodemographic status and IQ) and all analyses were covaried for previous own and parental psychiatric history. These factors may have confounded Kim *et al.*’s (2013) results and obscured any regulatory benefits conveyed by early CA. A second important distinction was the negative stimuli comprised pictures (Kim *et al.*, 2013) *vs* film clips (this study). The latter arguably elicit feeling states that are closer to the complex affective states experienced in everyday life compared with pictures, which have been shown to elicit brief and transient emotions (Hariri *et al.*, 2002; Britton *et al.*, 2006). These differences in design may account for additional variance in the findings and if supported by future research strengthen the findings from this study. Alternatively, Kim *et al.* (2013) may have found childhood poverty to have an adverse impact on ER capacity because it constitutes a more severe form of adversity than those experienced in this study. Indeed the systemic nature of poverty is likely to impact all areas of development, which in turn will affect the development of ER capacity.

The present results are, however, in line with the findings reviewed above showing individuals exposed to CA but who never developed depression to exhibit reduced ventrolateral PFC and dorsal ACC connectivity during the regulation of emotions to negative pictures.

The present findings are further consistent with an established evidence base in the rodent and non-human primate literatures, and other emerging evidence in humans, that moderate early life stress can promote processes important for resilience (Fergus and Zimmerman, 2005; Parker and Maestripieri, 2011; Seery *et al.*, 2013). This may occur by virtue of undesirable experiences leading to more frequent opportunities to learn and practice ER skills (and develop ER capacity), even though day-to-day ER application remains unpredictable and context sensitive. The challenge model of resilience proposes that early life stressors in the non-extreme range can act as potential ‘enhancer[s] of competence’ (Zolkoski and Bullock, 2012), which allow children to practice mobilizing their resources within the developmental process (Fergus and Zimmerman, 2005; Yates *et al.*, 2003). For instance, children exposed to a moderate or controlled pattern of adversities may actually show later benefits when faced with further environmental adversities (Rutter, 2006). An upside to adversity has also been shown experimentally, with reduced difficulties in task performance in individuals exposed to moderate cumulative adverse life events (Seery *et al.*, 2013).

It is important to emphasize, as noted in the ‘Introduction’ section, that better ER capacity as measured in the laboratory does not mean that the individual will experience fewer ER difficulties in day-to-day life. Indeed, as evidenced by the extensive literature linking self-reported and observer-rated ER problems in the day-to-day with early CA (Pechtel and Pizzagalli, 2010), it remains likely that differential family ecology and pressures endemic to early adversity will mean that such ER ‘application’ is impaired in those exposed to mCA relative to non-exposed peers but through more proximal environmental toxins (St Clair *et al.*, 2014). Moreover, due to the participants’ young age it is problematic to establish resilience. Systemic—potentially ongoing—environmental factors that were the categorized as CA may over-ride any potential benefits of ER capacity as measured in this study. This in turn could account for the finding that 44% of the mCA+ group showed a higher rate of previous psychiatric diagnoses. That is, improved ER capacity may only be beneficial in the context of ordinary environments where individuals occasionally face moderate levels of adversity.

Moreover, inferences to the overall ROOTS sample need to be drawn with caution given the selection of the present mCA group without current psychiatric disorder. It may be that ER does break down in those currently suffering from a psychiatric condition the etiology of which may be found in other pathogenic processes such as for example biased information processing (Mathews and MacLeod, 2005).

We also cannot conclude that enhanced ER capacity, as measured in the laboratory, will be associated with a history of severe CA, such as childhood physical or sexual abuse (Pechtel and Pizzagalli, 2010), as individuals experiencing these more severe forms of CA were not included in this study. This is a question for future research. In fact we hypothesize based on the extent literature on the pervasive adverse effects of these more severe forms of CA that ER will be reduced in these individuals. Specifically, as noted above we argue that severe forms of CA lead to numerous emotion processing deficits including attentional biases, increased emotional reactivity and reduced cognitive capacity all of which are likely to interact to reduce ER capacity. Given the potentially differential effects on ER, and possibly on cognitive-affective processes, future work would benefit from evaluating the effects of CA including samples spanning the full continuum of adversity.

It was interesting that, although mCA was associated with greater ER capacity in response to both positive and negative emotive stimuli in the behavioral data, the significant neural effects were limited to negative contexts. It is of course important not to over-interpret null findings. However, this pattern may indicate that the arguably enhanced neural efficiency associated with negative ER in those exposed to mCA does not extend to positive ER because there has been little opportunity to develop and consolidate positive ER skills. Instead, it may be that mCA simply provides those exposed with a richness of negative appraisals for potentially positive events, thus augmenting the capacity to reduce positive affect (the behavioral effect) without altering the underlying efficiency of the relevant neural substrates.

This study has a number of critical strengths. First, the study design allowed us to prospectively and longitudinally investigate the effect of mCA in the first 11 years of life, as assessed at age 14, on ER capacity at age 20. This contrasts with the majority of previous imaging studies, which have relied on retrospective recall of CA at the time of testing (for a review see Pechtel and Pizzagalli, 2010; however, it should be noted that in our sample recall of mCA was also retrospective though closer in time—recall at 14-years for CA before age 11 years).

Another strength of these findings comes from our ability to match the groups on a number of potential confounds including genetic, mood, cognitive, psychiatric and demographic variables through the selection of participants from a large representative cohort, and careful, interview-led evaluation of early adversity and psychiatric disorder.

A further strength is that our imaging analyses were carefully driven by the prior theoretical and empirical literature, being based on ROIs emerging in a previous meta-analysis on the process of interest under investigation in this study (Buhle *et al.*, 2014)—ER (defined as the cognitive reappraisal of a situation and the feelings elicited to reduce distress).

Finally, the present findings should be interpreted in the light of a number of limitations. First, participants may not have adhered to the ERT instructions. That is, they may have deployed strategies other than simply watching the film or reappraising its content. This was prevented as far as possible through extensive practice sessions where participants were comprehensively briefed and provided with examples of what constitutes reappraisal as well as asked to verbalize their regulation strategies during the practice session. Moreover, previous studies using this task have shown good compliance with the reappraisal instructions (Schweizer, 2012; Schweizer *et al.*, 2013). A further limitation is that maltreatment and trauma were not assessed beyond the age of 11 years. Traumatic events between 11 years of age and the age at testing may have impacted on the current findings. These later traumata may also be associated with some of the psychiatric disorders observed in this sample. Future research should be comparative in the effects of childhood *vs* early and mid-adolescent adversity on ER capacity in late adolescence and early adulthood. Related to the above point of later adversity is the fact that this study design did not allow for the comparison of potentially differential effects of adversity depending on the developmental stage in childhood. Future research should therefore extend the comparative scope by assessing whether the stage of development at which adversity occurred has differential effects on ER capacity. Finally, we evaluated our sample during the period of late adolescence/emerging adulthood—a period that is likely critical during the development of ER skills (Crowell *et al.*, 2013). However, this was to some extent determined by the constraints of the larger cohort study within which the current research is embedded (Goodyer *et al.*, 2010). There is a case for future studies to evaluate the evolution of ER capacity across adolescence, focusing on critically sensitive period defined by extant theoretical models.

In sum, this is the first study to our knowledge to investigate the longitudinal effects of any form of early CA on ER capacity, measured in the laboratory and its neural substrates. The data suggest that moderate CA is associated with elevated ER capacity for both positive and negative emotions and with enhanced efficiency in the neural networks underpinning the regulation of negative effect.

## Supplementary Material

Supplementary Data

## Footnotes

1 See Supplementary Table S1 for effect of condition on ROI activation.

2 A possible account for the absence of any effect during positive conditions may be that the selected ROIs bias toward the detection of effects for negative stimuli given that the studies included in the meta-analysis on which the ROIs were based primarily investigated the reappraisal of negative stimuli (Buhle *et al.*, 2014). We therefore supplemented our analyses for the positive conditions with whole-brain analyses of positive ER and reactivity. The full factorial analyses with condition (ER analysis: Positive Regulate *v**s* Positive Attend; emotional reactivity analysis: Positive Attend *v**s* Neutral Attend) as the within-subject factor and mCA exposure as the between-subject factor revealed no differential activation at *P*_FWE_ < 0.05 for either positive ER or reactivity.

3 Overall reactivity across valence showed no group differences either *F*(1,51) = 0.01, *P* = 0.935.
