# Supplementary material for: Enhanced emotion regulation capacity and its neural substrates in those exposed to moderate childhood adversity
Source: Soc Cogn Affect Neurosci. 2015 Sep 4;11(2):272–81. doi: 10.1093/scan/nsv109 (PMC4733337; doi:10.1093/scan/nsv109)
Supplement: Supplementary Data [file supp_11_2_272__index.html]

Enhanced emotion regulation capacity, and its neural substrates in those exposed to moderate childhood adversity — Enhanced emotion regulation capacity and its neural substrates in those exposed to moderate childhood adversity — Enhanced emotion regulation capacity and its neural substrates in those exposed to moderate childhood adversity — Supplementary Data 

# Enhanced emotion regulation capacity and its neural substrates in those exposed to moderate childhood adversity

## Supplementary Data

files

- Supplementary Data - docx file
