# Supplementary material for: Enhanced emotion regulation capacity and its neural substrates in those exposed to moderate childhood adversity
Source: Soc Cogn Affect Neurosci. 2015 Sep 4;11(2):272–81. doi: 10.1093/scan/nsv109 (PMC4733337; doi:10.1093/scan/nsv109)
Supplement: Supplementary Data [file supp_nsv109_scan-15-105-File003.docx]

**Supplementary Materials**

Enhanced emotion regulation capacity, and its neural substrates, in those exposed to moderate childhood adversity

Susanne Schweizer

Nicholas Walsh

Jason Stretton

Valerie Dunn

Ian M. Goodyer

Tim Dalgleish

Included

Supplementary Introduction

Supplementary Methods

Figure S1

Supplementary Results

Table S1

# Supplementary introduction

## The effects of CA on amygdala and PFC

The amygdala, which is essential to emotion processing and emotional learning of adaptive behavioural and physiological responses to threat (Whalen & Phelps, 2009), is typically found to be enlarged in individuals reporting severe childhood adversity, with amygdala volumes showing a dose-response relation to adversity duration (e.g., Tottenham et al., 2010). That is, adversity appears to impact on the development of the neural substrates of emotional reactivity. Accordingly, functional magnetic resonance imaging (fMRI) shows potentiated amygdala reactivity to threat-related stimuli in individuals exposed to severe adversity compared to unexposed peers (Bogdan, Williamson, & Hariri, In press; Tottenham et al., 2011). It has been proposed that heightened amygdala reactivity to emotional stimuli, especially faces, is a consequence of emotional learning of their relative survival value (Tottenham et al., 2011; however see: Walsh et al., 2012). Rather than compromising ER capabilities themselves it may be that adversity instead sensitises emotional reactivity, testifying to the importance of measuring emotional reactivity *per se* in our experimental paradigm, as well as ER.

A second line of evidence shows that significant early adversity affects prefrontal cortical development. Adults with a history of CA show reduced grey matter volumes in the PFC, especially the medial PFC and anterior cingulate cortex (ACC) (for reviews see: McCrory, De Brito, & Viding, 2011; Tomoda et al., 2009; Treadway et al., 2009; van Harmelen et al., 2010). The lateral and medial PFC, including the dorsal and subgenual ACC (sgACC), constitutes the primary neural substrate of ER (Ochsner & Gross, 2005; for reviews see: Phillips, Ladouceur, & Drevets, 2008; Wager, Davidson, Hughes, Lindquist, & Ochsner, 2008). These same regions show reduced grey matter volumes and are hypoactivated in psychiatric disorders characterised by emotion dysregulation (Davidson et al., 2002; Price & Drevets, 2012; Rauch et al., 2003; Shin et al., 2005). These imaging findings then are in line with the large body of work showing CA to be associated with a range of negative outcomes later in life including cognitive and affective functioning and psychopathology (Kessler et al., 2010; Pechtel and Pizzagalli, 2010; Read and Bentall, 2012).

# Supplementary methods

## Assessment of Childhood Adversity (0-11 years) and parental psychiatric history

The CAMEEI, a semi-structured interview, is conducted with the child’s primary caregiver and records family-focused adverse life experiences, child’s age at occurrence, duration, and an interviewer assessment of their practical impact on the daily life of the family (Dunn et al., 2011). The MINI Mental State Examination (Sheehan et al., 1997) was embedded within the CAMEEI to assess current and lifetime parental mental illness.

## Genotyping: DNA collection, extraction and analysis

DNA was harvested from separate saliva samples (Qiagen, Crawley, UK) and genotyped for 5-HTTLPR. The 5-HTTLPR region was amplified using the primers 5-ATGCCAGCACCTAACCCCTAATGT-3 and 5-GGACCGCAAGGTGGGCGGGA-3, which generates a 419bp and 375bp product for the “l” and “s” alleles respectively. The PCR reaction mixture consisted of: 100ng genomic DNA, 10mM Tris-HCl (pH 9.0), 1.5mM MgCl_2_, 50mM KCl, 0.1% Triton®X-100, 1.25U *Taq* DNA polymerase, 200μM dNTPs, 500nM each of forward and reverse primer and 100μM 7-Deaza-dGTP in a final reaction volume of 15μL. The reaction conditions were 98°C for 7 mins, followed by 40 cycles of 96°C for 30 secs, 61°C for 30 secs and 72°C for 1 min with a final extension stage of 72°C for 10 mins. PCR products were electrophoresed on a 3700 DNA analyser (Applied Biosystems) with semi-automated sizing and genotyping performed using GENESCAN v3.7 and GENOTYPER v3.7 software (Applied Biosystems).

## Neuroimaging data acquisition

A 3T Siemens Tim Trio MRI scanner was used to collect 745 echoplanar imaging (EPI) volumes. All EPI data had 32 slices, matrix size of 64 x 64, echo time (TE) of 30 msecs, repetition time (TR) of 2 secs, field of view of 19.2x19.2 cm, flip angle of 78°, slice thickness of 3 mm, interslice distance of .75 mm, and in-plane resolution of 3x3 mm. High-resolution magnetization-prepared rapid-acquisition gradient echo (MPRAGE) anatomical images (TR of 2250 ms, TE of 2.99 ms, flip angle of 9°, inversion time of 900 ms, 256x240x192 isotropic 1 mm voxels) were collected for anatomic localization and coregistration.

Images were sinc-interpolated in time to correct for acquisition time differences and realigned spatially with respect to the first image using trilinear interpolation. The coregistered MPRAGE image was segmented and normalized using affine and smoothly nonlinear transformations to the T1 template in Montreal Neurological Institute (MNI) space. The normalization parameters were then applied to the EPIs, and all normalized EPI images were spatially smoothed with a Gaussian kernel of full-width half maximum 8 mm.

SPM5 was used for data analysis. For each participant and ERT condition, event types were modelled separately using a regressor made from an on–off boxcar convolved with a canonical hemodynamic response function. Six estimated parameters of movement between scans (translation and rotation along x, y, and z axes) were entered as covariates of no interest. Before running the model, the time course of the average brain signal was screened for spikes of high variance. Short periods of high variance are usually associated with brief subject movements as shown in the spatial realignment parameters. The high-variance scans were removed from the model by using a modified version of the SPM99 modelling routines (for details see: www.mrc-cbu.cam.ac.uk/Imaging/Common/missing_times.html). Low-frequency noise was removed with a standard high-pass filter of 120 sec. The results estimated from single subject models were entered into second-level random effects analyses for standard SPM group inference (Penny et al., 2003).

## Figure S1. Regions of interest included in the present study


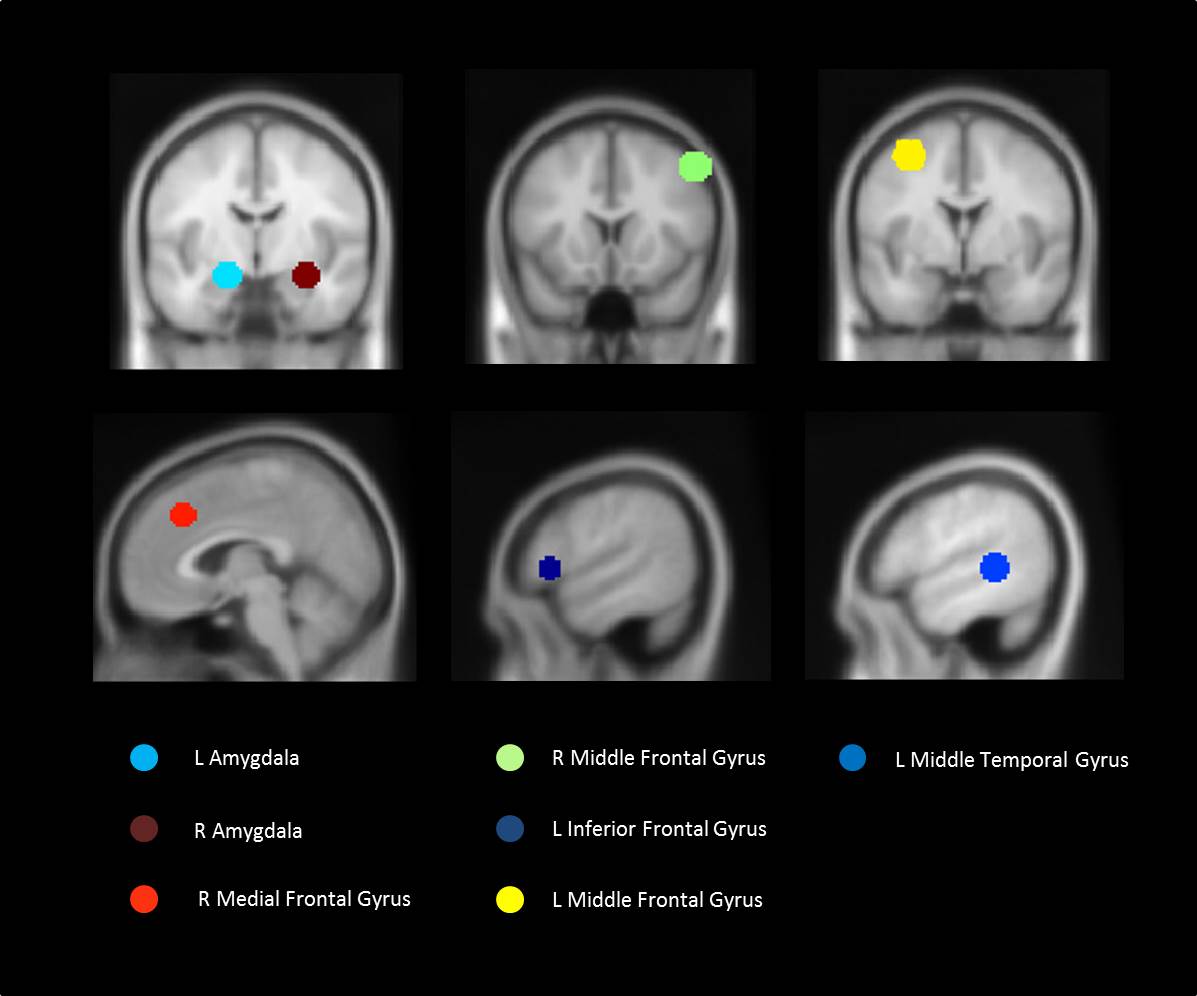


# Supplementary results

The multivariate test of condition was significant *F* (7, 28) = 2.99, *p* = .004, η_p_^2^ = 0.78. See Table S1 for univariate comparisons

## Table S1. Effects of condition on ROI activation

| ROI | L/R | Negative Reactivity | Positive Reactivity | Negative Regulation | Positive Regulation |
| --- | --- | --- | --- | --- | --- |
|  |  | *∆M* (*SEM*) | *∆M* (*SEM*) | *∆M* (*SEM*) | *∆M* (*SEM*) |
| Inferior frontal gyrus | R | 0.13 (0.09) | 0.01 (0.07) | −0.16 (0.08)^†^ | −0.01 (0.07) |
| Middle frontal gyrus | L | −0.01 (0.06) | −0.03 (0.05) | −0.07 (0.04) | −0.02 (0.04) |
|  | R | 0.09 (0.07) | 0.07 (0.05) | −0.06 (0.06) | 0.01 (0.05) |
| Medial frontal gyrus | R | 0.05 (0.06) | 0.06 (0.05) | −0.03 (0.05) | −0.04 (0.04) |
| Middle temporal gyrus | L | 0.12* (0.04) | 0.02 (0.04) | −0.11* (0.04) | 0.10* (0.03) |
| Amygdala | L | 0.10 (0.05)^†^ | −0.01 (0.05) | −0.06 (0.04) | 0.05 (0.03) |
|  | R | 0.10 (0.06)^†^ | 0.00 (0.05) | −0.08 (0.05)^†^ | 0.06 (0.03)^†^ |

Negative Reactivity = Negative Attend – Neutral Attend; Positive Reactivity = Positive Attend – Neutral Attend; Negative Regulate = Negative Regulate – Negative Attend; Positive Regulate = Positive Regulate – Positive Attend; *∆M* = mean difference in average BOLD activation across the ROI; *SEM* = standard error of the mean; L = left; R = right. ^†^ *p* < .10, * *p* < .05.
